# Study on optimization of layout parameters of high-level boreholes in Pingdingshan coal mine

**DOI:** 10.1038/s41598-023-46280-z

**Published:** 2023-11-13

**Authors:** Yapeng Wang, Yongli Zhang

**Affiliations:** 1 College of Innovation and Practice, Liaoning Technical University, Fuxin, 123000 China; 2 Science Foundation Management Office, Liaoning Technical University, Fuxin, 123000 China

**Keywords:** Computational science, Scientific data, Software

## Abstract

To reduce the gas disaster of high gas coal seam and improve the efficiency of gas extraction by high drilling, the layout parameters of drilling holes in Pingdingshan coal mine are optimized. Based on the analysis and calculation of the "three zones" of the movement towards the overly strata of No.10 coal in Pingdingshan coal mine, the height of caving zone and fissure zone in 24,130 working face are 10.06–14.46 m and 38.75–49.95 m respectively. The elevation angle, azimuth angle and the length of high-level boreholes are studied and analyzed by COMSOL numerical simulation software. The simulation results show that the optimum layout parameters of high-level boreholes are as follows: The elevation angle of borehole should be controlled at 9°–12°, the azimuth angle should be 30°–45°, and the length of borehole should be 150 m. Then the optimum layout parameters of high-level boreholes are determined for engineering application of 24,130 working face. Borehole data onto actual mine show that the optimum layout parameters of high-level boreholes were elevation angle between 8°and 11°, azimuth angle between 30° and 42°, and length of boreholes between 145 and 155 m. The simulation results are basically consistent with the measured data. The maximum gas concentration in working face, upper corner and return air roadway is stably controlled below 1%. The safe mining of 24,130 working face is ensured, which provided a certain reference value of gas control in the goaf of Pingdingshan mine and adjacent mines.

## Introduction

After underground mining starts, a large number of cracks will be generated in the fracture zone of the goaf, which is an important channel for gas flow migration^1–3^. If the gas extraction borehole is located in the above interval, the gas extraction effect will be significantly improved^4–8^. If the layout of high level boreholes is not reasonable, extraction blind areas will be formed, which will affect the effect of gas extraction^9–14^. Therefore, the reasonable layout of high level borehole determines the effect of gas extraction.

Taking Huaibei Qingdong coal mine as the research object, Liu et al.^15–17^ established the pressure relief and permeability enhancement model of gob drilling, simulated the mathematical relationship between the permeability of coal seam samples and the overburden gas pressure, and determined that pressure relief has a significant anti-outburst effect. Yang et al.^18,19^ studied the mechanism of coal seam water injection and pressure relief and reflection enhancement from the perspective of coal microstructure. Qu et al.^20^ studied the key factors affecting gas drainage in longwall horizontal goaf and concluded that the gas drainage effect depends on borehole location and reservoir conditions. Kan et al.^21^ firstly introduced the pumping principle of the high level borehole, then analyzed the factors affecting the goaf gas drainage, and finally determined the placement position and parameters of the high level borehole in Xiaoqing Mine, which has important reference significance for the determination and optimization of the subsequent gas drainage. Wang et al.^22^ proposed a new gas drainage method for pressure relief gas drainage by using drilling and large depth drilling through rocks and coal seams, which was applied in Haizi coal mine and finally made the gas drainage rate as high as 73%. Karacan et al.^23^ studied gas drainage borehole patterns at different heights and conducted a "dynamic" 3D reservoir modeling for a 381 m wide longwall coal wall in Pittsburgh coal seam. The results show that two—and three-sided drilling is more effective in reducing emissions. Hao et al.^24^ used rfpa2d integration to simulate the development of rock fractures and gas rich areas, and better solved the problem of gas emission in goaf. Esterhuizen et al.^25^ studied the gas production model of goaf gas drainage hole, simulated and analyzed the influence of overburden gas migration on mining environment by numerical simulation, and finally optimized the gas extraction method of goaf. Zhao et al. optimized the parameters of deep hole shaped charge blasting in Pingdingshan coal mine, and determined the optimal blasting hole aperture, charge position and charge length, which has guiding significance for the application of shaped charge blasting technology in Pingdingshan. The above-mentioned scholars primarily analyzed specific measures to improve gas drainage efficiency by examining the factors influencing gas migration in goaf areas, without further discussing the impact of of the difference of the Angle and location of the actual borehole on gas drainage from fractured zones in the goaf.

Therefore, this study focuses on the actual situation of the 10# coal seam in Pingdingshan coal mine, using numerical simulation software to determine the range of high-level borehole arrangement based on comprehensive parameters including elevation angle, azimuth angle, and borehole length. Field validation was conducted in the roadway of the 24,130 working face in Pingdingshan Coal Mine, and the measured data from the field were found to be consistent with the simulation results. Finally, the optimal layout parameters for high-level boreholes in the 24,130 working face of Pingdingshan coal mine were determined.

## Prediction of the height of “three zones” of overlying strata fractures

Using the empirical formula method, the calculation of the height of the caving zone for the 24,130 fully-mechanized mining working face is performed^26^. Based on the geological survey data of Pingdingshan coal mine, the immediate roof of the 10# coal seam mainly consists of mudstone and sandy mudstone, with some areas comprising siltstone and fine to medium-grained sandstone. According to the measured results of rock mechanics parameters, the average compressive strength of the mudstone is 27.5 MPa, and the average coal thickness is 5.5 m. Therefore, the calculation is performed based on the assumption of a medium-hard rock layer:

1 $$ H_{m} = \frac{100\sum M }{{4.7\sum {M + 19} }} \pm 2.2 = \frac{100 \times 5.5}{{4.7 \times 5.5 + 19}} \pm 2.2 = 10.06m\sim 14.46m $$

where *M* is the cumulative thickness of the mined coal seam, m (it is taken as 5.5 m); *H*_*m*_ is the Height of the caving zone, m.

To calculate the height of the fractured zone within the 24,130 gas drainage working face, the immediate roof of the 10# coal seam is typically composed of K2 limestone. Therefore, it can be treated as a hard rock layer for calculation purposes.

2 $$ H_{l} = \frac{100\sum M }{{1.6\sum {M + 3.6} }} \pm 5.6 = \frac{100 \times 5.5}{{1.6 \times 5.5 + 3.6}} \pm 5.6 = 38.75m\sim 49.95m $$

## Numerical simulation of in-situ borehole gas drainage

### Construction of numerical calculation model

To determine the impact of parameters such as elevation angle, azimuth angle (angle with respect to the tunnel wall), and borehole length on gas drainage effectiveness, this study utilizes the COMSOL numerical simulation software based on the specific conditions of the site. A coupled model is constructed considering the coal seam occurrence conditions and extraction parameters. The variation in gas concentration is used as the indicator for optimal borehole arrangement. By studying the various influencing factors, the optimal layout parameters for high-level boreholes are determined.

**Figure 1:**
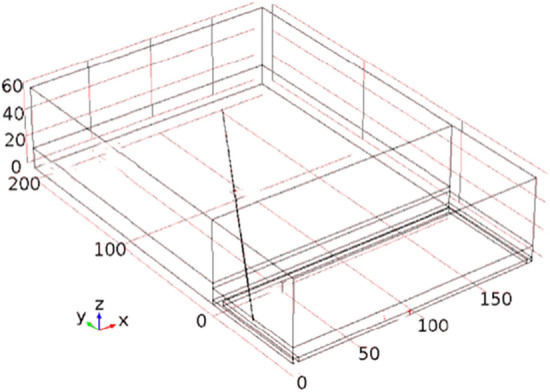
The geometric model of high-level borehole gas drainage.

**Figure 2:**
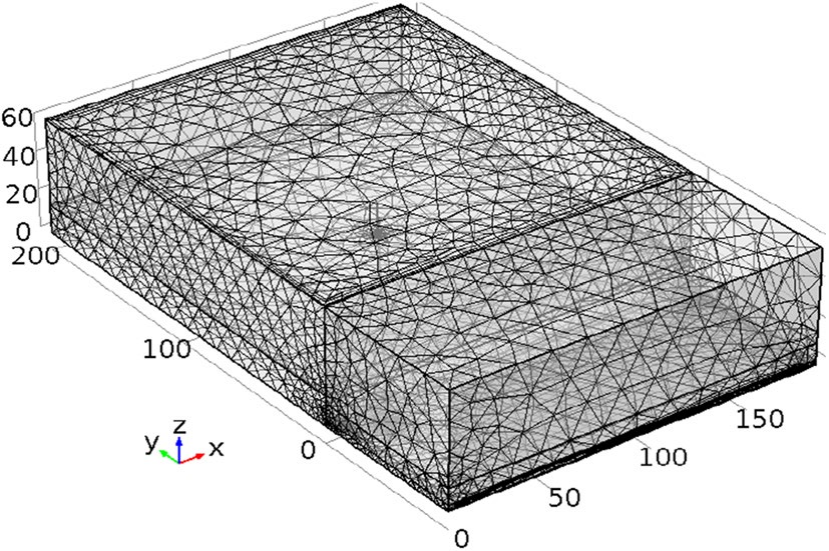
Meshing of the model.

Based on the actual parameters of the high-level boreholes in the 24,130 working face of Pingdingshan coal mine, this study selects the following parameters: inclined length of the working face is 186 m, goaf length is 200 m, dimensions of the intake and return airways are 3 × 4 m, height of the working face is 3 m, width is 5 m, height of the caving zone is 15 m, height of the fractured zone is 45 m, length of unmined solid coal behind the working face is 80 m, and coal thickness in the solid coal region is 11.5 m. The overlying strata above the coal seam in the unmined area are considered as rock layers. The geometric model is shown in Fig. 1. The meshing of the model is shown in Fig. 2. The elevation angle of the borehole is denoted as *a*_1_, the angle between the borehole and the coal seam is denoted as *a*_2_, and the length of the borehole is L. Due to the significant computational workload of three-dimensional models, this study only simulates individual boreholes and does not consider the interaction between boreholes. The main focus is to optimize the borehole elevation angle, azimuth angle, and length as key technical indicators through simulation.

### Numerical model parameters and boundary conditions

The numerical simulation in this paper is based on the relevant physical parameters of Pingdingshan coal mine. The inlet speed is controlled at 2.5 m/s, the air volume is 1800 m^3^/min, the return air outlet is controlled by the pressure difference between ends 110 Pa, the high level borehole is set as the mass outflow boundary, the extraction flow rate is *q*_ch4_, the solid coal is set according to the original gas content, and the goaf is set according to the proportion of residual gas. The height of the fracture zone is 45 m, and the height of the collapse zone is 15 m. The relevant parameters are shown in Table 1.

**Table 1:** Physical parameters of porous media.

Parameter name	Parameter symbol	Value and unit
Young's modulus	*Ee*	2.713e9[Pa]
Poisson's ratio	*Mu*	0.339
Coal density	*Densc*	1250 [kg/m^3^]
Gas density	*Densg*	0.717 [kg/m^3^]
Dynamic viscosity	*Visco*	1.84e−5 [Pa·s]
Langmuir constant	*PL*	6.019e6 [Pa]
Langmuir volume strain constant	*Sorpl*	0.02295
Initial porosity of coal	*Phi0*	0.084
Initial permeability of coal	*K0*	2e−14 [m^2^]
Raw coal gas pressure	*Pi0*	0.5e6 [Pa]
Negative pressure extraction	*Pa*	20000[Pa]

### Analysis of simulation results of influence of high level borehole parameters on extraction effect

#### The effect of elevation angle of borehole

**Figure 3:**
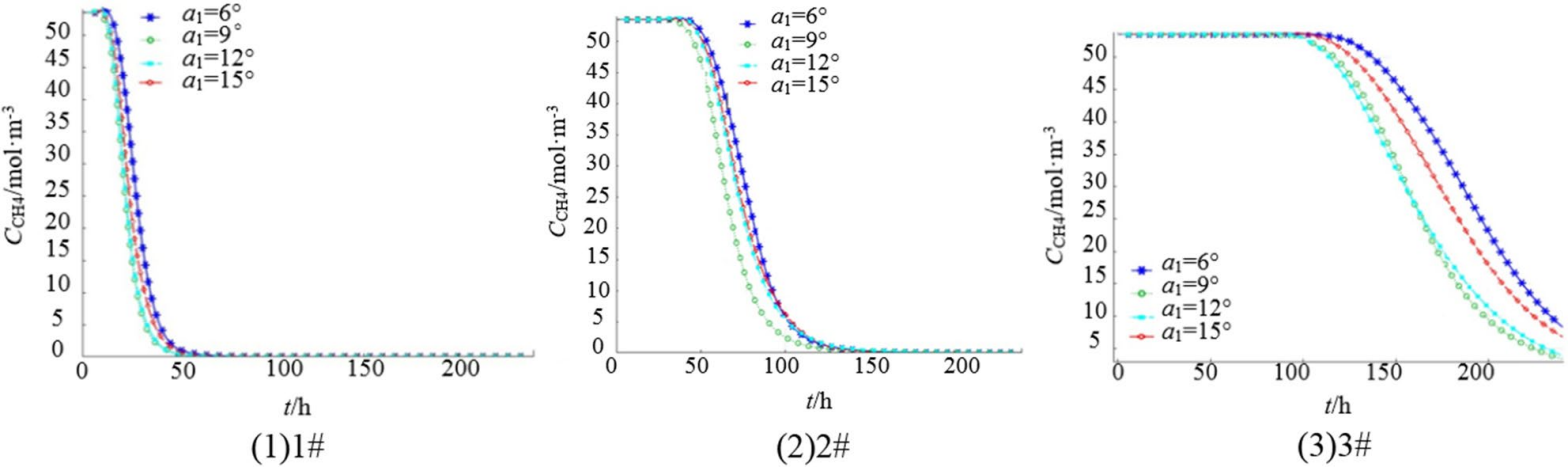
Variation curve of gas concentration at each monitoring point under different elevation angle.

The elevation angle of a single borehole is set as *a*_1_ = 6°,9°,12°, and 15°, borehole length L = 150 m, angle between borehole and coal wall *a*_1_ = 30°, extraction flow *q*_ch4_ = 3 m^3^/min, and extraction time is 240 h. Figure 3 shows the change of gas concentration at 1#(93,50,30), 2#(93,100,30) and 3#(93,150,30) monitoring points in the goaf.

Figure 3 shows that the gas concentration at 1# starts to decrease significantly after 12 h of extraction, and gas extraction is completed after 50 h. Among the tested elevation angles, the lowest residual gas concentration is observed at an elevation angle of *a*_1_ = 12°, which is nearly equivalent to that at *a*_1_ = 9°. The highest residual gas concentration is observed at *a*_1_ = 6°, followed by *a*_1_ = 15°. The gas concentration at 2# begins to decline notably after 40 h of extraction, and gas extraction is completed after 140 h. Among the tested elevation angles, the lowest residual gas concentration is observed at *a*_1_ = 9°, while the highest concentration is observed at *a*_1_ = 6°, and *a*_1_ = 15° yields the second-highest concentration. The gas concentration at 3# starts to decrease significantly after 100 h of extraction, and there is still a certain concentration of residual gas after 240 h. Among the tested elevation angles, the lowest residual gas concentration is observed at *a*_1_ = 9°, followed by *a*_1_ = 12°, while the highest concentration is observed at *a*_1_ = 6°.

**Figure 4:**
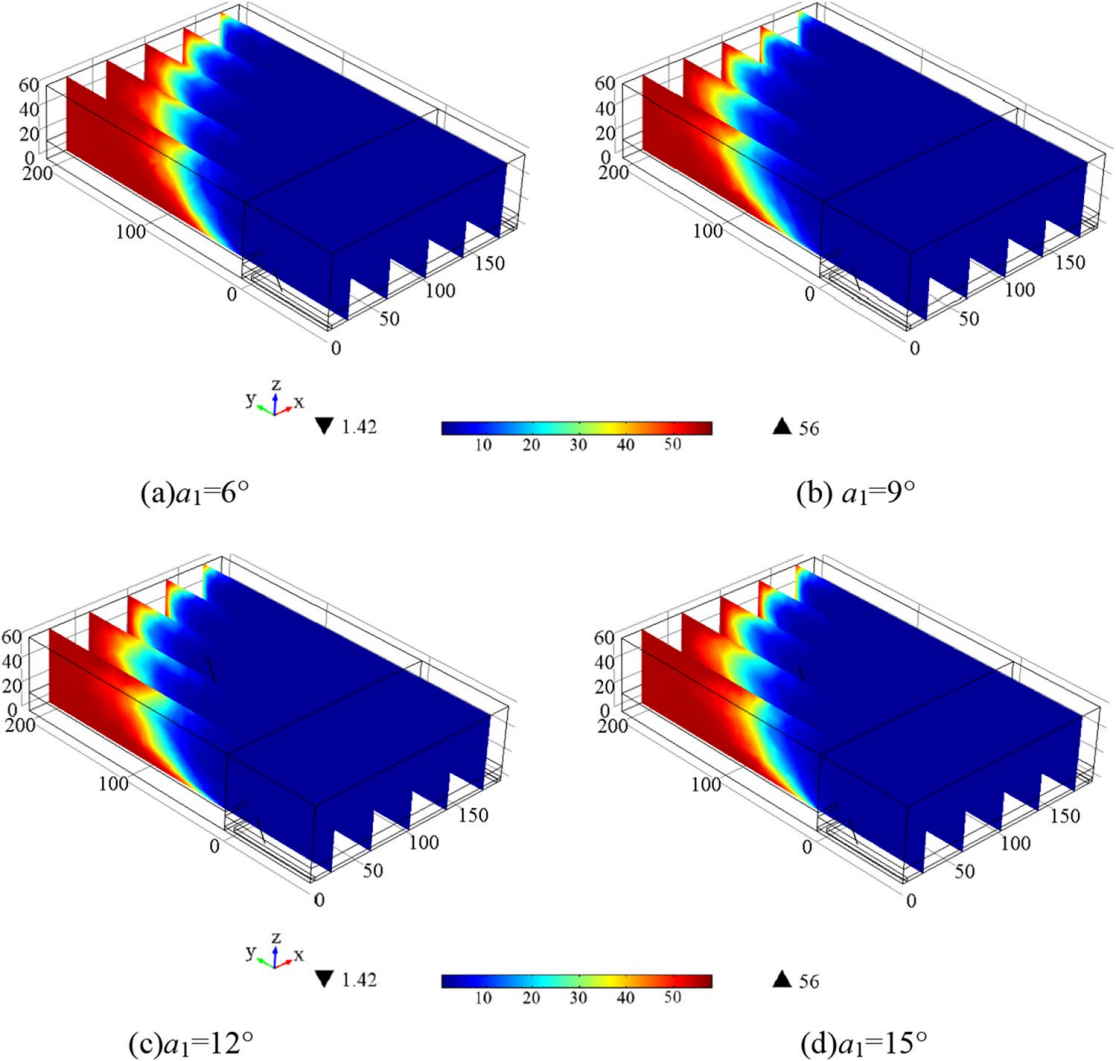
Gas concentration distribution in 240 h extraction under different *a*_1_.

Figure 4 shows that the gas concentration is relatively high at *a*_1_ = 6°, 15°, highest when *a*_1_ = 6°, and second-highest when *a*_1_ = 15°. The concentration is relatively lower at *a*_1_ = 9° or 12°, lowest when *a*_1_ = 9°, and second-lowest when *a*_1_ = 12°. This suggests that the optimal range for the elevation angle* a*_1_ is greater than 6° and should be maintained between 9°and 12° for better gas extraction effectiveness.

#### The effect of borehole azimuth angle

**Figure 5:**
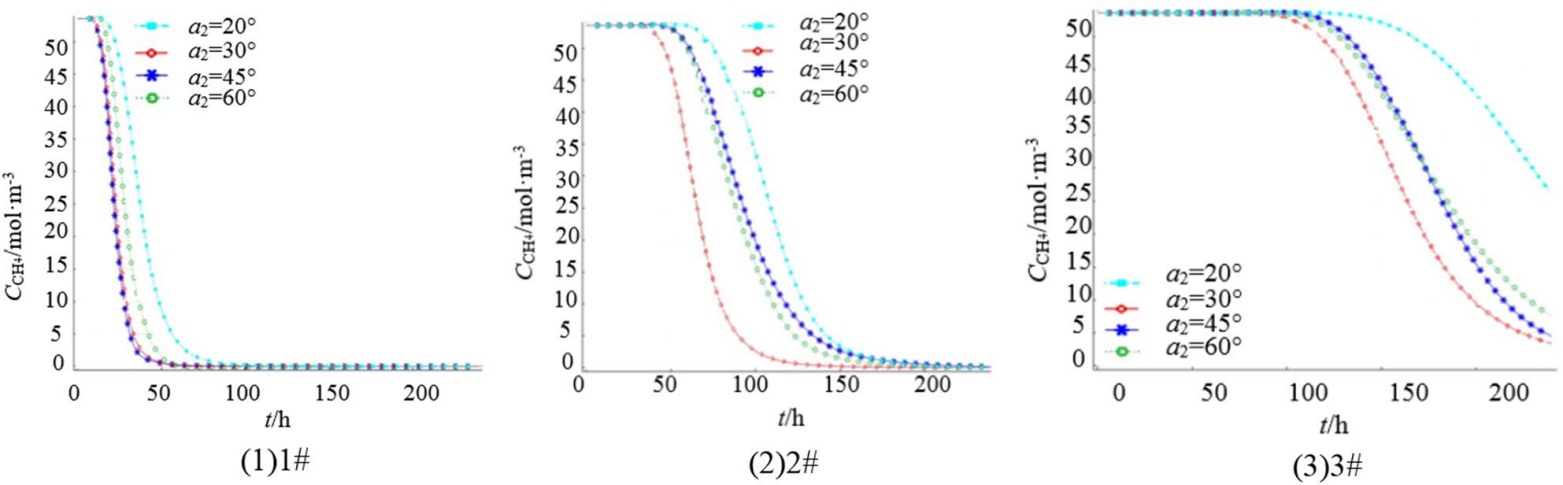
Variation curve of gas concentration at each monitoring point under different borehole azimuth.

Set the azimuth angle of each borehole to 20°, 30°, 45°, and 60°, length of borehole *L* = 150 m, elevation Angle of borehole *a*_1_ = 9°, and extraction time to 240 h. Figure 5 shows the change of gas concentration at 3 monitoring points of 1#–3# in the goaf. Figure 5 shows that the gas concentration at 1# began to decrease significantly after 6 h of extraction, and the gas extraction is completed after 80 h, in which the residual gas concentration is the lowest when the azimuth Angle *a*_2_ = 30° and 45°, the highest when *a*_2_ = 20°, and the second when *a*_2_ = 60°. The gas concentration at 2# began to decrease significantly 42 h after extraction, and is completed 180 h after extraction. The lowest concentration of residual gas is found at *a*_2_ = 30°, the highest concentration is found at *a*_2_ = 20°, followed by *a*_2_ = 45 and 60°. After 100 h of extraction, the gas concentration at 3# began to decrease significantly, and after 240 h of extraction, there is still a certain concentration of gas. The highest concentration of residual gas is found when *a*_2_ = 20°, the lowest concentration is found when *a*_2_ = 30°, followed by *a*_2_ = 45 and 60°, indicating that the azimuth between 30° and 45° is the best effect.

**Figure 6:**
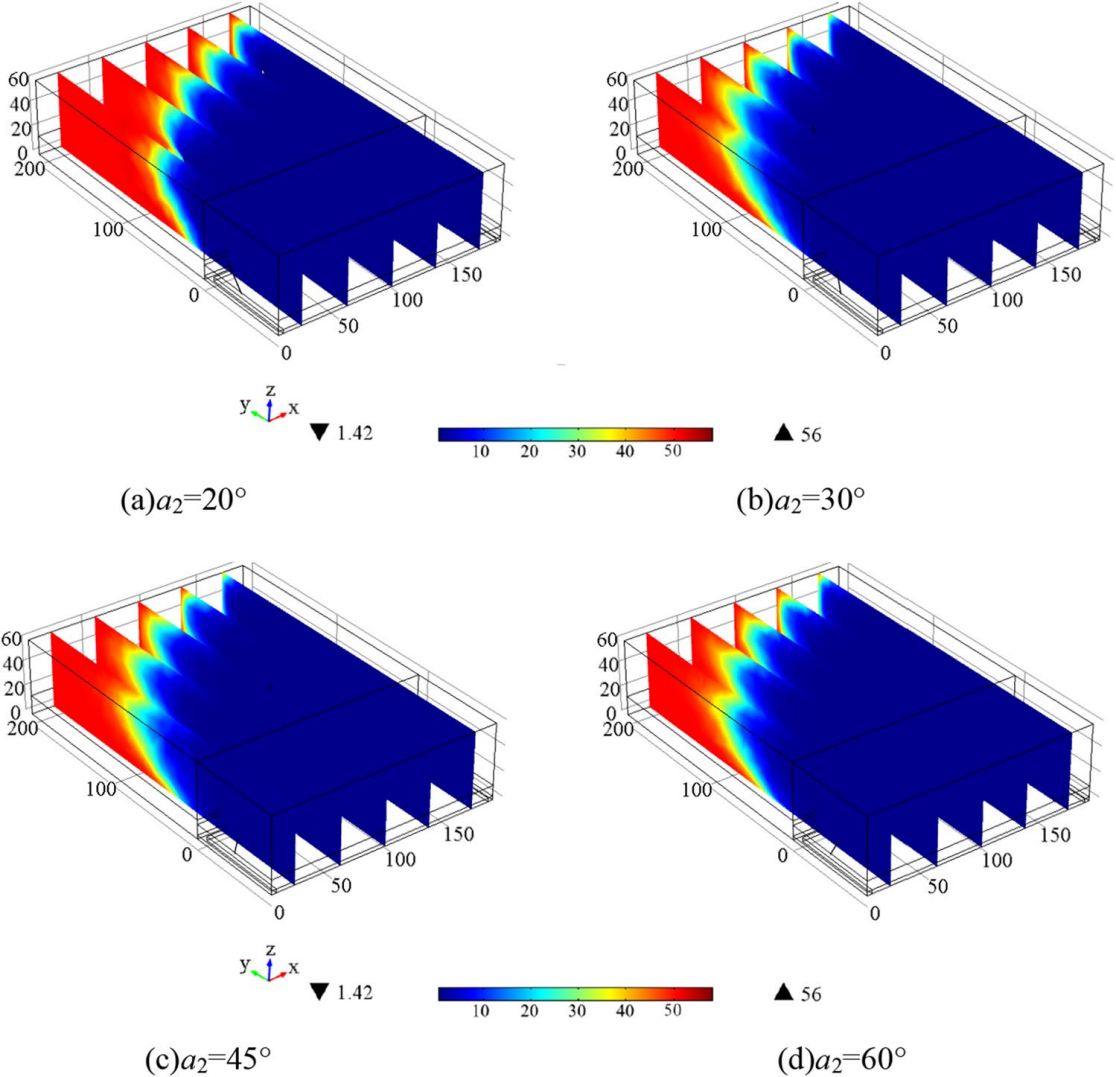
Gas concentration distribution in 240 h extraction under different *a*_2_.

Figure 6 displays the gas concentration distribution in 240 h extraction under different *a*_2._ The figure shows that the residual gas content is lower in the intake air side and higher in the return air side. Additionally, when *a*_2_ is between 30° and 45°, the residual gas content throughout the goaf is relatively low, indicating a more significant gas extraction effect and better gas extraction efficiency.

#### The effect of borehole length

**Figure 7:**
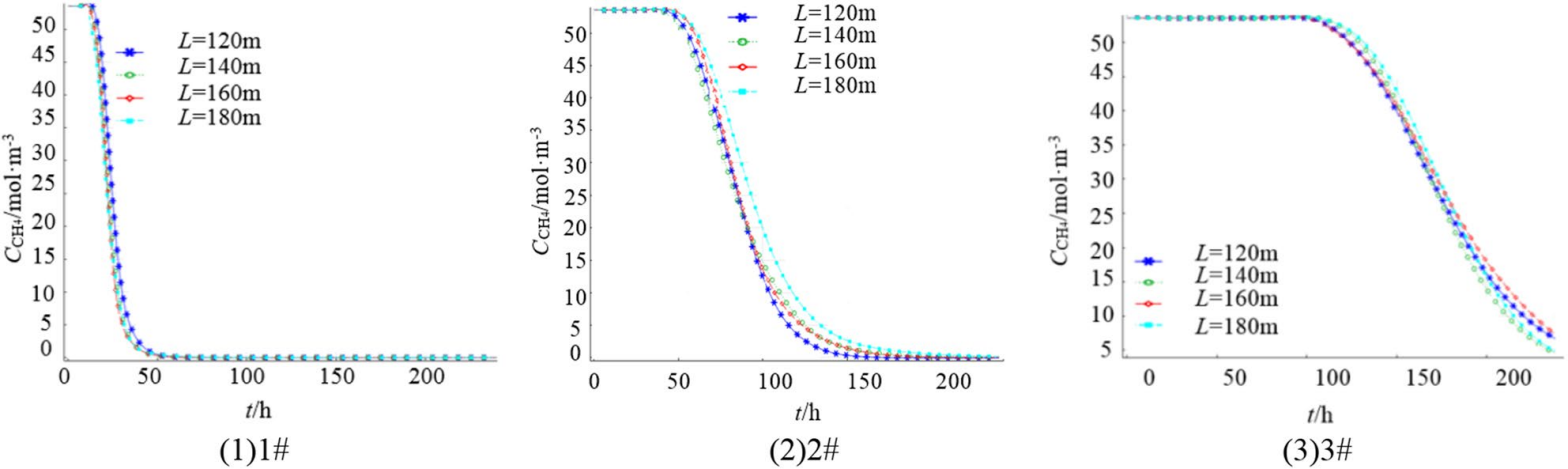
Variation curve of gas concentration at each monitoring point under different borehole length.

For each individual borehole, the lengths are set as *L* = 120 m, 140 m, 160 m, and 180 m, with an elevation angle of *a*_1_ = 9°, azimuth angle of *a*_2_ = 30°, gas extraction flow rate of *q*_ch4_ = 3m^3^/min, and a total extraction time of 240 h. Figure 7 illustrate the gas concentration variations at three monitoring points.

Figure 7 demonstrates that the residual gas content in the shallow part of the goaf has a low correlation with the length of the borehole, as varying the borehole length does not result in significant differences in residual gas content. At 2#, the highest residual gas content in the goaf is observed when *L* = 180 m, while the residual gas content gradually decreases for *L* = 140 m, 160 m, and 120 m. In the deeper part of the goaf, there is minimal variation in the trend of residual gas content for *L* = 120 m, 140 m, 180 m, and 120 m. In conclusion, under unchanged conditions, the borehole should not be too short and should be greater than 120 m to ensure a significant influence range in the horizontal direction. Controlling the length within the range of 140–160 m leads to lower residual gas content in the goaf and relatively better gas extraction effectiveness.

**Figure 8:**
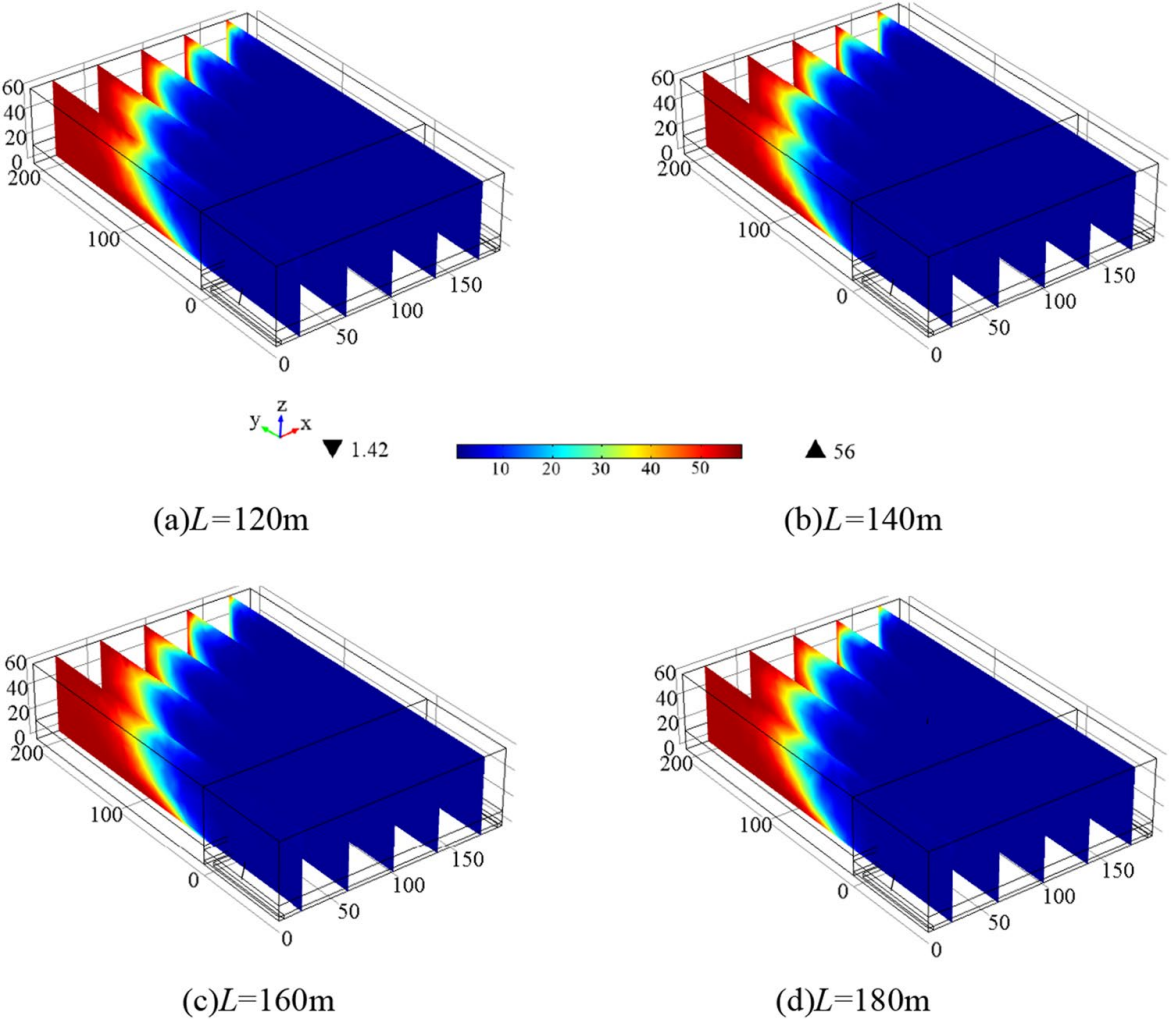
Gas concentration distribution in 240 h extraction under different *L.*

Figure 8 presents the gas concentration distribution in the goaf after 240 h of extraction under different borehole lengths. The figure illustrates that the larger the borehole length, the lower the residual gas content in the goaf. However, when the length reaches 180 m, the decrease in residual gas content in the goaf is not significant.

## Field industrial test

### Test site profile

The 24,130 working face in Pingdingshan coal mine is located at an elevation of + 860 to + 936 m. It has a strike length of 1113 m and a dip length of 220 m, covering an area of 244860 m^2^. The mine has an absolute gas emission rate of 57.26 m^3^/min and a relative gas emission rate of 26.8 m^3^/min. The coal seam in this working face has a dip angle ranging from 0° to 5°, with an average thickness of 3.5 m. The coal seam has a bulk density of 1.46 t/m^3^ and a permeability of 0.42 m^2^/(MPa^2^ d), indicating that it belongs to a high-gas-emitting coal seam.

### Test drilling site and drilling construction design

According to the theory of mining ellipsoid zone in overburden rock, when the fully mechanized caving face is fully influenced by the initial and periodic mining processes, the middle of the caving face is compacted, and a certain width mining fracture zone is formed on both sides, and the mining fracture field shows a parabolic distribution in the inclined direction. The layout of high directional long boreholes should be considered from the horizontal and vertical distance, and should be arranged in the fracture zone of high gas enrichment area with dense distribution of horizontal and vertical fractures, so as to ensure the extraction effect.

In general, the horizontal distance from the compaction area above the goaf to the roadway is less than the initial pressing step. Based on the research on the change rule of overlying rock cracks and gas migration rule in the goaf and the analysis of numerical simulation, three sets of high level boreholes are finally arranged to further improve the extraction effect of high level boreholes.

Determining drilling field spacing is an important means to ensure effective length lap between drilling holes, which can ensure efficient extraction, effective utilization, length and drilling field spacing of drilling holes. The calculation process is shown in Formula (3–5).

3 $$ \rho = \frac{{H{}_{k} - H_{m} }}{{H_{k} }} \times 100\% $$

4 $$ L_{{\text{s}}} = L\cos a_{1} $$

5 $$ N = 6.67 \times \frac{{Q^{2} }}{{\pi \times d^{2} \times V \times C}} $$

where $\rho$ is drilling efficiency, %; *H*_*k*_ is the height of the borehole, m; *L*_*s*_ is drilling field spacing, m; *L* is the length of the hole, m; *N* is the number of high level holes; *Q* is the total extraction purity of the drilling field, m^3^/min; *d* is the diameter of drilling hole, mm; *V* is the gas flow velocity, m/s; *C* is the extracted gas concentration, %.

**Figure 9:**
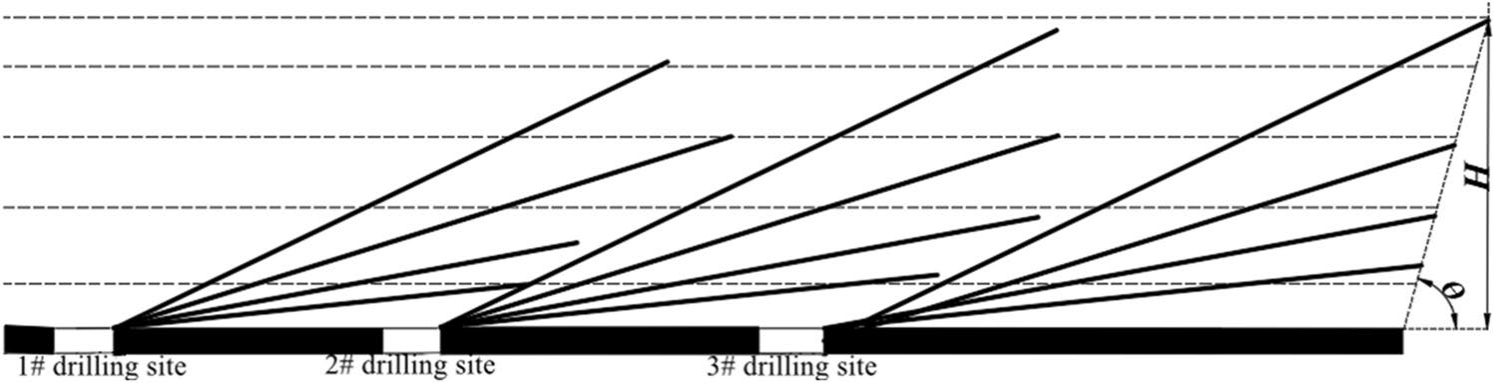
Schematic diagram of the drilling construction at the test site.

According to the caving zone and fracture zone calculated in "Introduction" section and formula (3–5), it is determined that the single-hole pumping flow rate is 4 m^3^/min, four high-level drilling holes are constructed in each high-level drilling field, and three test high-level drilling fields are arranged in the working face of 24,130. The distance between the first high-level drilling field and the cutting hole is. The spacing between the drilling fields of the high level drilling holes is to ensure that the drilling stubble is above. Figure 9 shows the layout diagram of high extraction borehole. Detailed drilling layout parameters of the three high-position drilling fields are shown in Table 2.

**Table 2:** Drilling parameters.

Drilling number	Borehole azimuth *α*_*2*_ (°)	Elevation angle *α*_*1*_ (°)	Length *L* (m)	Diameter *d* (mm)	Horizontal distance *X* (m)	Vertical distance *H* (m)
1	30	5	150	113	130.5	13.1
2	30	8	150	113	131.2	20.9
3	30	11	150	113	132.3	28.6
4	30	14	150	113	133.5	36.3
5	30	9	150	113	131.5	23.5
6	25	9	150	113	137.7	23.5
7	42	9	150	113	112.8	23.5
8	58	9	150	113	80.5	23.5
9	30	9	145	113	127.1	22.7
10	30	9	165	113	144.7	25.8
11	30	9	155	113	136.0	24.2
12	30	9	175	113	153.4	27.4

### Analysis of gas extraction effect

**Figure 10:**
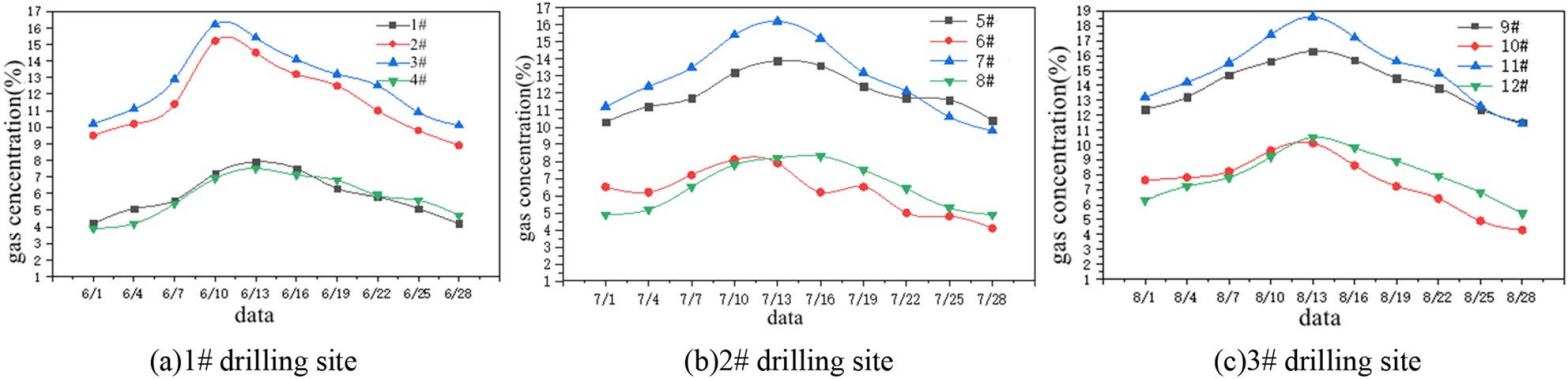
Variation curve of gas concentration in drilling site.

The proposed scheme was implemented for the later-stage gas extraction in the 24,130 working face, and the volume fraction of extracted gas was continuously monitored for a period of 3 months. The changes in gas volume fraction at the working face, return airway, and upper corner are summarized in Fig. 10.

According to Fig. 10, with the continuous advancement of the working face, the net amount of gas extracted from the upper borehole shows a trend of first increasing and then decreasing. By comparing the extraction concentrations of the four boreholes in Fig. 10a, it can be found that the average gas concentrations of boreholes 2# and 3# are 11.68% and 12.72% respectively, while the average values of boreholes 1# and 4# are 5.89% and 5.26% respectively, and the average gas concentrations of boreholes 2# and 3# are 6.63% higher than those of boreholes 1# and 4#. It can be seen that when the Angle and length of the drilling hole remain unchanged, the dip Angle is 8°–11°, and the pumping effect is better when the Angle is between. It can be seen from Fig. 10b that the average gas extraction values of boreholes 5# and 7# are 10.92% and 13.24% respectively, while the average values of boreholes 6# and 8# are 6.21% and 6.13% respectively, and the average gas concentration values of boreholes 5# and 7# are 5.91% higher than those of boreholes 6# and 8#. The gas extraction effect is better when the Angle of borehole is between 30°and 42° when the inclination and length of borehole remain unchanged. As can be seen from Fig. 10c, the average gas extraction values of boreholes 9# and 11# are 13.05% and 15.47% respectively, while the average values of boreholes 10# and 12# are 6.02% and 6.79% respectively, and the average gas concentration values of boreholes 9# and 11# are 7.86% higher than those of boreholes 10# and 12#. The gas extraction effect of boreholes 9# and 11# is obviously greater than that of boreholes 10# and 12#. It can be seen that when the Angle and inclination of boreholes remain unchanged, the extraction effect is better when the length of boreholes is between 145 and 155 m. Based on the above analysis, the optimal layout parameters for high-level boreholes are an inclination angle between 8° and 11°, an included angle between 30° and 42°, and a length between 145 and 155 m.

As can be seen from Table 3, numerical simulation results show that the optimal extraction borehole parameters are: dip Angle 9°–12°, included Angle 30°–45°, and borehole length 140–160 m. Field experimental results show that the optimal extraction borehole parameters are: The dip Angle is 8°–11°, the included Angle is 30°–42°, and the drilling length is 145–155 m. It can be seen that the field measured results are basically consistent with the numerical simulation data, which verifies the validity of the numerical model.

**Table 3:** Comparison of results.

Comparison of optimal gas extraction position results	Numerical simulation results	Field measurement result
Elevation angle	9°–12°	8°–11°
Azimuth angle	30°–45°	30°–42°
Borehole length	140–160 m	145–155 m

**Figure 11:**
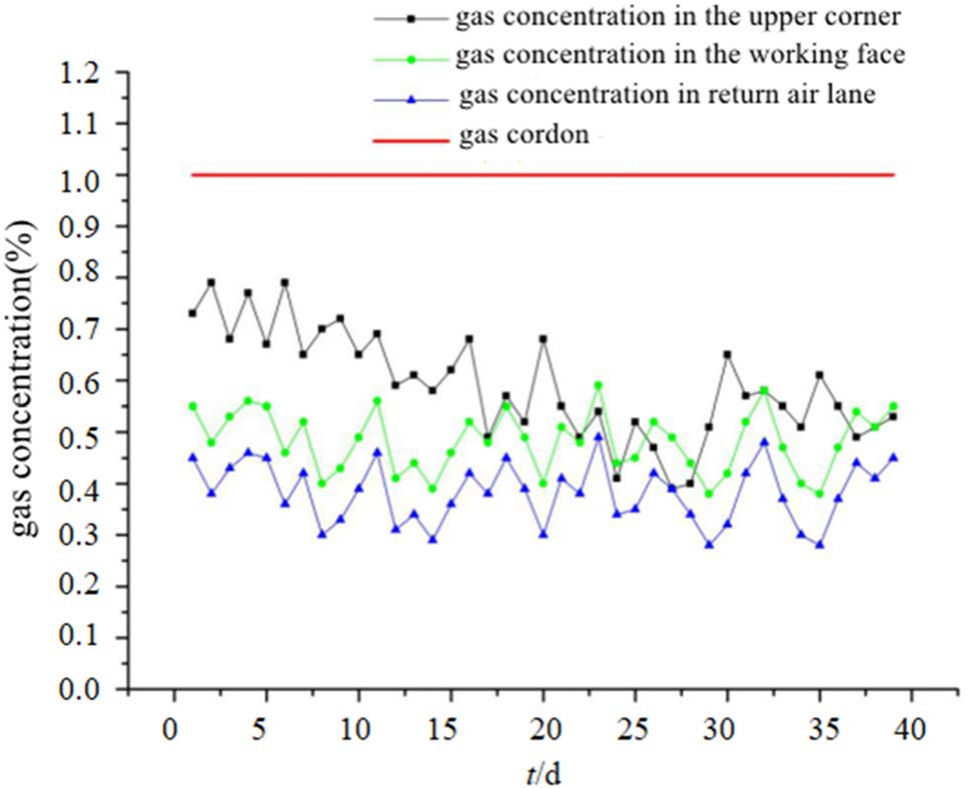
Gas volume fraction change curve.

By conducting real-time observations of the gas volume fractions in the mining face, upper corner angle, and return airway during gas extraction at drill sites 1#, 2#, and 3#, the changes in gas volume fractions in the mining face, upper corner angle, and return airway are shown in Fig. 11. As can be seen from Fig. 11, the gas extraction effect of the high drilling is significant, and the average gas concentration of the working face is 0.54% and the maximum gas concentration is 0.61%. The average gas concentration in the upper corner is 0.69%, and the maximum gas concentration is 0.8%. The average gas concentration in the return air roadway is 0.41%, and the maximum gas concentration is 0.55%, which is far lower than the maximum gas volume fraction 1% stipulated in the Coal mine Safety Regulations, and ensures the efficient production during the working face.

## Result and discussion

Based on the analysis of the scope of the hidden area of gas accumulation in the upper part of 24,130 working face (the height of roof caving zone and caving fracture zone), the layout scheme of gas extraction borehole in the upper part of the working face is optimized from the Angle, Angle and length of borehole, which provides the test data basis for the design of gas extraction borehole in the upper part of the working face. The results of numerical simulation and field experiment show that the optimal layout parameters of the high drilling hole are the inclination of 8°–11°, the included Angle of 30°–42°, and the drilling length of 145–155 m. After the optimization of drilling fields 1#, 2# and 3#, the average gas extraction concentration of drilling holes is significantly increased, the gas concentration of working face, upper corner and return air roadway is significantly reduced, and the gas treatment effect of goaf is significantly improved.

## Conclusion

- (1) The analysis and calculation have determined that the height of the goaf zone in the 24,130 mining face is between 10.06 and 14.46 m, and the fracture zone height is between 38.75 and 49.95 m. This provides a theoretical basis for the establishment of a numerical simulation model for high-level borehole gas extraction
- (2) Based on the relevant physical parameters of Pingdingshan coal mine, a numerical simulation of in-situ borehole gas extraction was conducted, and the simulation results were analyzed. The results indicate that when the inclination angle of the borehole is controlled between 9° and 12°, the azimuth angle is controlled between 30° and 45°, and the borehole length is 150 m, the gas extraction effect of the fracture zone in the goaf is optimal
- (3) By investigating the extraction volume at different layouts of high-level boreholes and combining it with field measurements, the optimal placement position of the borehole's terminal point was determined. According to the field data, the optimal borehole layout parameters are an inclination angle between 8° and 11°, an azimuth angle between 30° and 42°, and a borehole length between 145 and 155 m, which is consistent with the simulation results
- (4) Through the monitoring of gas conditions in the mining face, upper corner angle, and return airway, it has been found that the maximum gas concentration in the 24,130 mining face and associated areas remains stable and controlled below 1%. The high-level borehole gas extraction has achieved the expected effect in the goaf gas, ensuring the safe and smooth mining of the working face.

## Data Availability

The datasets used and/or analysed during the current study available from the corresponding author on reasonable request.
